# Letter to the editor: Addressing the blind spot of self-reported adherence in the era of long-acting PrEP

**DOI:** 10.2807/1560-7917.ES.2026.31.2.2500974

**Published:** 2026-01-15

**Authors:** Roberto Brunoro, Daniele Mengato, Francesco Barbaro, Lucrezia Calandrino, Anna Maria Cattelan, Francesca Venturini

**Affiliations:** 1 University of Milan, Milan, Italy; 2 Padua University Hospital, Hospital Pharmacy Unit, Padua, Italy; 3 Padua University Hospital, Infectious and Tropical Diseases Unit, Padua, Italy

**Keywords:** HIV, pre-exposure prophylaxis, PrEP, long-acting injectable, cabotegravir, CAB-LA, adherence

**To the editor:** We read with great interest the rapid communication by Moschese et al. on the early implementation of long-acting injectable cabotegravir (CAB-LA) for HIV pre-exposure prophylaxis (PrEP) in Italy [1]. The authors report promising data on the feasibility and high adherence rates of CAB-LA in a real-world setting, specifically targeting individuals with barriers to oral PrEP. As one of the prescribing centres for CAB-LA in Italy, we fully support the authors' conclusions. Our real-world experience aligns with their findings, confirming that long-acting formulations are a feasible and highly accepted option for HIV prevention in Italy, effectively addressing many challenges associated with daily oral intake [2]. However, as the implementation of CAB-LA expands, accurately identifying individuals who experience challenges with oral adherence — one of the key eligibility criteria cited by Moschese et al. — is crucial for prioritising this resource effectively.

In this context, we would like to highlight a methodological challenge we encountered at the University Hospital of Padua: the difficulty of accurately quantifying adherence when relying solely on patient self-reporting. At our centre, we introduced a digital version of the prescription form issued by the Italian Medicines Agency (AIFA) to simplify the prescribing process and enhance pharmacist-led monitoring. This tool, while initially procedural, allowed us to cross-reference self-reported data with objective pharmacy refill records for a cohort of 611 individuals, as recently presented [2]. Our analysis revealed a notable discrepancy between perceived and actual adherence. Among users on a continuous daily regimen who self-reported 100% adherence during clinical interviews, pharmacy-based monitoring using the proportion of days covered (PDC) indicated that 14.5% (29/200) had a PDC < 80%, indicating suboptimal coverage. Furthermore, we identified a subset of users (13/200) with a PDC > 120%, suggesting potential informal sharing of medication, a phenomenon undetectable through self-reporting alone [2].

In this specific regard, CAB-LA offers a structural advantage beyond adherence: by administering the drug directly in the clinical setting, the risk of incorrect oral therapy intake is eliminated. This ensures that the therapy effectively protects the intended user while implicitly encouraging others to access formal care channels rather than relying on shared pills. These findings underscore a blind spot in standard assessments. Had we relied solely on the interview, nearly 15% of users with suboptimal protection — ideal candidates for the CAB-LA strategy described by Moschese et al. — might have been misclassified as adherent and overlooked for prioritisation. Conversely, objective pharmacy data can reveal the true candidates who would benefit most from long-acting options. 

Additionally, beyond clinical targeting, our experience highlights a logistical consideration. As demonstrated by Moschese et al., implementing CAB-LA requires robust operational protocols. Our findings suggest that digitising the prescription process substantially streamlined workflows and reduced administrative burden [2]. We argue that adopting such digital infrastructure is essential for centres aiming to scale up long-acting programs sustainably, allowing staff to reallocate time from administrative tasks to the enhanced counselling and monitoring that injectable therapies require.

We agree with Moschese et al. that CAB-LA is a crucial tool for closing prevention gaps. To maximise its public health impact, we suggest that clinical programs should combine objective adherence measures, such as digital pharmacy refill tracking with patient self-reports. This dual approach ensures eligibility criteria are applied accurately and directs long-acting agents to those who will benefit most.
